# The development of emotional intelligence in childhood: associations with general developmental abilities

**DOI:** 10.3389/fpsyg.2026.1999245

**Published:** 2026-10-23

**Authors:** Rosario Cabello, Manuel Pérez-López, Desirée Llamas-Díaz, María José Gutiérrez-Cobo, Vicenta María Ruiz-Baeza, Pablo Fernández-Berrocal

**Affiliations:** 1 Department of Developmental and Educational Psychology, Faculty of Psychology, University of Málaga, Málaga, Spain; 2 Department of Basic Psychology, Faculty of Psychology, University of Málaga, Málaga, Spain

**Keywords:** age, childhood, development, emotional intelligence, general abilities

## Abstract

Emotional intelligence (EI) is thought to develop alongside broader cognitive, communicative, and motor abilities during childhood. However, evidence from early childhood remains limited, particularly when EI is assessed using performance-based measures. This cross-sectional study examined age- and gender-related differences in EI and general developmental abilities, as well as the associations between them, in 377 Spanish children aged 48–83 months (*M* = 71.82, *SD* = 7.45). Emotion perception, understanding, and management were assessed using the Emotional Intelligence Test of the Botín Foundation for Children, a performance-based measure, while cognitive, communication, and motor abilities were assessed using the Battelle Developmental Inventory (BDI). We examined gender differences, bivariate associations, linear and quadratic age trends, and multivariable predictors of each EI ability. Girls scored higher than boys in emotion perception, whereas boys scored higher in cognitive abilities, although both differences were small. No gender differences emerged in the remaining abilities. Age was positively associated with all general developmental and EI abilities, although these associations were stronger for general developmental abilities. Quadratic age terms produced only trivial increases in explained variance, supporting a predominantly linear pattern across the age range examined. General developmental abilities were positively, albeit modestly, associated with EI. In the final regression models, cognitive ability independently predicted emotion perception (*β* = 0.177, *p* = 0.004) and, most strongly, emotion management (*β* = 0.322, *p* < 0.001), whereas communication ability independently predicted emotion understanding (*β* = 0.181, *p* = 0.002). Age retained a small independent association only with emotion perception, while motor ability made no unique contribution. The final models explained 8.1%, 4.4%, and 9.6% of the variance in emotion perception, understanding, and management, respectively. Taken together, these findings suggest that performance-based EI abilities form part of broader developmental processes while showing distinct associations with cognitive and communication abilities. They also highlight the potential value of integrating emotional learning with developmentally appropriate cognitive and communication activities in early educational settings to support children’s socio-emotional development.

## 1 Introduction

Traditionally, research on child development has examined its components relatively independently, treating them as discrete domains. Although this approach has enabled the detailed investigation of individual domains, it has limited our understanding of how different developmental processes interact (Denham, 1998). More recently, however, interest in integrative approaches that conceptualise development more broadly has grown. Rather than considering developmental domains in isolation, research increasingly examines the relationships among them. For example, some studies have explored the association between cognitive and language development (Pu et al., 2025), while others have investigated the interplay between cognitive and motor skills (Bao et al., 2024; Van Der Fels et al., 2015) or between language and motor skills (González et al., 2019; Rhemtulla and Tucker-Drob, 2011). Taken together, this evidence highlights the interdependent nature of developmental processes and the importance of considering multiple domains simultaneously when studying child development.

Regarding the relationship between these domains and emotional development, research conducted since the 1980s has sought to identify the most important emotional competences and clarify how they relate to broader human development (Denham, 1986). Emotional competence refers to the ability to understand, regulate, and express emotions in ways appropriate to the social context (Denham, 1998). Research indicates that emotional understanding and regulation—rather than emotional expression alone—play a key role in developing social competence during the preschool years (Denham et al., 2003). Children who apply greater emotional knowledge in emotionally charged situations demonstrate significant advantages in interactions with their peers: they respond more prosocially, are perceived by their teachers as more socially competent, and are better liked by their peers (Denham, 1986, 1998; Denham and Couchoud, 1990; Denham et al., 2003). Moreover, these abilities not only facilitate social adjustment but are also consistently associated with better academic performance (Denham et al., 2012; Trentacosta and Izard, 2007).

In the 1990s, emotional intelligence (EI) emerged as a theoretical model integrating core emotional abilities and their development, enabling a more comprehensive examination of emotional competence. According to the ability model proposed by Mayer and Salovey (1997), EI is defined as ‘the ability to accurately perceive, appraise, and express emotion; the ability to access and/or generate feelings that facilitate thought; the ability to understand emotions and emotional knowledge; and the ability to regulate emotions to promote both emotional and intellectual growth’ (p. 10). This study is grounded in the theoretical model of EI established by Mayer and Salovey (1997) and later developed by Mayer et al. (2016).

The ability model identifies two main approaches to assessing EI. The first uses objective measures that assess how well individuals solve emotion-related problems. Notable examples include the Mayer-Salovey-Caruso Emotional Intelligence Test (MSCEIT; Mayer et al., 2002) and the Emotional Intelligence Test of the Botín Foundation for Adolescents (TIEFBA; Fernández-Berrocal et al., 2018b). The second approach uses self-report or informant-report measures to assess individuals’ subjective perceptions of their own EI or others’. One example is the Trait Meta-Mood Scale (TMMS; Joseph and Newman, 2010). Performance-based assessments require participants to solve emotional problems, and responses can be evaluated as correct or incorrect, making them comparatively more objective than self-report measures. By contrast, self-report measures capture individuals’ perceptions of their own emotional abilities and may therefore be influenced by social desirability bias. Performance-based measures, meanwhile, tend to predict behaviour more consistently (Brackett et al., 2006; Gutiérrez-Cobo et al., 2016; Webb et al., 2013). Although both approaches assess EI, correlations between them are generally low, suggesting they capture distinct aspects of the construct. Using both approaches may therefore provide a more comprehensive understanding of EI (Brackett and Mayer, 2003).

Previous research has consistently demonstrated that EI is associated with beneficial outcomes across the lifespan. Specifically, higher EI has been associated with better mental health, subjective well-being, academic performance, personal adjustment, and empathy, as well as lower levels of aggressive behaviour and substance misuse and fewer suicide attempts (Balluerka et al., 2013; Cabello et al., 2017; Davis and Humphrey, 2012; Domínguez-García and Fernández-Berrocal, 2018; Fernández-Berrocal et al., 2018b; Gomez-Baya et al., 2016; Kun and Demetrovics, 2010; Llamas-Díaz et al., 2022; MacCann et al., 2020; Vega et al., 2022). However, research that adopts an integrative approach to examining emotional development, such as the EI framework, in relation to other developmental domains during early childhood remains scarce. Some studies involving primary school pupils have examined the relationship between EI and performance indicators, particularly reading and overall academic performance, and have reported positive associations (MacCann et al., 2020; Yu et al., 2023). However, most of these studies have assessed EI using self-report measures grounded in mixed models. Only one study has examined this relationship using an objective, performance-based measure derived from the EI ability model (Rivers et al., 2012). Rivers et al. (2012) conducted a cross-sectional study involving adolescents aged 10 to 13 years. They found that higher EI scores were significantly associated with healthier psychological functioning, greater social competence, and improved academic performance in English language arts. These positive relationships remained significant even after controlling for the participants’ verbal abilities. In addition, no studies have focused specifically on the early childhood education stage, likely because, until a few years ago, adequate instruments to assess EI as an ability at these ages were lacking (Fernández-Berrocal et al., 2018a).

This present study addresses four key challenges in investigating the development of EI during childhood. First, there is a lack of objective methods for evaluating EI in children aged 4 to 6 years. To address this challenge, we used the Emotional Intelligence Test of the Botín Foundation for Children (TIEFBI), which was specifically designed to assess EI as an ability in young children (Fernández-Berrocal et al., 2018a). The TIEFBI comprises three tests based on the EI ability model: *Catch the Emotion*, *In Search of the Lost Emotion*, and *The Emotional Detective*. A notable advantage of this instrument is its hands-on, visual format, in which emotions and situations are represented through illustrations and vignettes. This format makes the instrument more accessible to young children, whose language skills are still developing. The test is administered individually and takes approximately 30 min to complete.

The second challenge concerns age-related differences in EI. Research suggests that EI varies across the lifespan, following an inverted-U pattern during adulthood (Cabello et al., 2016b). During adolescence, EI appears to increase linearly, although this pattern has been observed only among girls (Megías-Robles et al., 2024). Although EI is expected to increase progressively during childhood, the timing and nature of this developmental trajectory have yet to be established empirically.

The third challenge is to examine gender differences in EI and general developmental abilities during early childhood. Previous studies have found that girls and women tend to demonstrate higher levels of ability-based EI than boys and men during adolescence and adulthood, respectively (Cabello et al., 2017; Cabello et al., 2016b; Megías-Robles et al., 2024). However, to our knowledge, no previous research has specifically examined gender differences in ability-based EI during early childhood. Nevertheless, research on emotion regulation in children suggests that girls generally exhibit better emotion-regulation abilities than boys (Liao and Lin, 2025).

The fourth challenge is to determine how EI relates to general developmental abilities—specifically cognitive, communicative, and motor abilities—in children aged 4 to 6 years. Recent research using early childhood developmental assessments has identified a general developmental factor underlying these three domains across several inventories, including the Battelle Developmental Inventory (BDI) (Trudel et al., 2026). This finding underscores the importance of assessing cognitive, communication, and motor abilities when interpreting early childhood development. Examining these domains alongside EI supports an integrative approach that addresses the fragmented view of development characteristic of much previous research.

In summary, this study had three primary objectives: (a) to examine gender differences in EI and general developmental abilities among children aged 4–6 years; (b) to investigate age-related patterns in EI and general developmental abilities; and (c) to examine the associations between general developmental abilities and EI.

Based on the literature reviewed, we formulated the following hypotheses: (H1) girls would obtain higher EI scores than boys; (H2) general developmental abilities (cognitive, communication, and motor abilities) would be positively associated with age; (H3) EI abilities (perceiving, understanding, and managing emotions) would be positively associated with age; and (H4) general developmental abilities would be positively associated with EI.

## 2 Methods

### 2.1 Participants

Participants were a community sample of 377 Spanish children recruited from six public nursery and primary schools across Málaga. The sample comprised 172 boys (45.6%) and 205 girls (54.4%). Participants ranged in age from 48 to 83 months (*M* = 71.82, *SD* = 7.45). Each child was assessed individually by a member of the research team using the TIEFBI and the Battelle Developmental Inventory. Responses were recorded either on paper or electronically via the LimeSurvey platform (https://www.limesurvey.org). Assessments were conducted in a quiet, comfortable, distraction-free room at each child’s school to provide consistent testing conditions.

### 2.2 Procedure

Participants were recruited using non-probability convenience sampling, based on school accessibility and participant availability. Specific inclusion and exclusion criteria were established to define the sample. The inclusion criteria were: (a) enrolment in early childhood education; (b) written informed consent from a parent or legal guardian; and (c) the ability to understand the tasks and follow instructions appropriate to the child’s educational level. Children were excluded if they had specific educational needs requiring continuous individualised support that prevented them from completing the assessments under standard administration conditions.

We conducted the study in accordance with the Declaration of Helsinki (World Medical Association, 2013). Because all participants were minors, we obtained written informed consent from their parents or legal guardians before data collection, ensuring confidentiality and adherence to current ethical standards. The study protocol was approved by the Research Ethics Committee of the University of Granada (2,498/CEIH/2021).

### 2.3 Instruments

The *Emotional Intelligence Test of the Botín Foundation for Children* (TIEFBI; Fernández-Berrocal et al., 2018a) is a performance-based measure of EI designed for children aged 2 years to 11 years and 11 months. Based on Mayer and Salovey’s (1997) ability model, it assesses emotion perception, understanding, and management through three tests. *Catch the Emotion* assesses the ability to identify basic emotions from photographs of children’s faces. *In Search of the Lost Emotion* assesses the ability to identify the emotions experienced by the protagonist in a series of stories. Finally, *The Emotional Detective* assesses the ability to select the most appropriate emotion-regulation strategy for a given situation. Standardised scores (*M* = 100, *SD* = 15) are calculated for each test. The instrument is administered individually and takes approximately 30 min to complete. In the present sample, the internal-consistency coefficients were as follows: perceiving (Cronbach’s α = 0.89; McDonald’s ω = 0.90), understanding (α = 0.70; ω = 0.67), and managing (α = 0.81; ω = 0.80).

The *Battelle Developmental Inventory* (BDI; Newborg, 2005; Newborg et al., 1984) is a norm-referenced instrument designed to assess the developmental status of children from birth to 7 years and 11 months. It includes five domains: adaptive, personal–social, communication, motor, and cognitive development. Standardised scores (*M* = 100, *SD* = 15) are calculated for each domain, with lower scores indicating greater developmental delay. The present study focused on the cognitive, communication, and motor domains, following the recommendations of Trudel et al. (2026).

The BDI identifies developmental strengths and weaknesses through structured activities and observations of the child’s behaviour. It is administered individually and is estimated to take 20 min per participant. Items are scored on a three-point scale ranging from 0 (the child does not demonstrate the skill) to 2 (the child fully demonstrates the skill). Previous research has shown the instrument’s reliability and internal consistency (Alfonso et al., 2010; Newborg, 2005). In the present sample, internal consistency was adequate across the three domains assessed: cognitive (α = 0.83; ω = 0.87), communication (α = 0.86; ω = 0.89), and motor (α = 0.88; ω = 0.92).

### 2.4 Data analysis

First, descriptive statistics were calculated for all study variables. Independent-samples *t-*tests were then conducted to examine gender differences in the general developmental domains and EI abilities, with Cohen’s *d* used to quantify effect sizes. Second, Pearson’s correlations were calculated to examine associations between age and the general developmental domains and EI abilities. Linear and quadratic regression models were also fitted to determine whether these associations were better characterised by linear or quadratic age trends. Third, Pearson’s correlations were used to examine the associations between the general developmental domains and EI abilities. Hierarchical stepwise regression analyses were then conducted separately for each EI ability to determine the unique contributions of the general developmental domains alongside age and gender. All analyses were performed using IBM SPSS Statistics, Version 30.

## 3 Results

Table 1 presents descriptive statistics for the study variables and the results of the independent-samples *t*-tests examining gender differences. Boys obtained significantly higher cognitive scores than girls, whereas girls obtained significantly higher emotion-perception scores than boys. Both differences were small in magnitude. No significant gender differences were found for the remaining variables.

**Table 1:** Descriptive statistics for the total sample and by gender (mean and standard deviation [SD]) and *t*-tests of gender differences (*t*-value and Cohen’s *d* effect size).

				Gender differences	
Domain/ability	Total sample mean (SD)	Boys mean (SD)	Girls mean (SD)	*t*	Cohen’s *d*
Cognitive	106.02 (8.70)	107.27 (8.55)	104.97 (8.71)	2.58**	0.27
Communication	105.82 (11.80)	105.84 (11.85)	105.80 (11.78)	0.03	0.00
Motor	107.48 (8.30)	107.06 (8.62)	107.83 (8.03)	−0.90	−0.09
Perceiving	102.98 (12.89)	101.34 (12.74)	104.35 (12.88)	−2.27*	−0.24
Understanding	100.22 (14.55)	100.68 (14.76)	99.83 (14.40)	0.56	0.06
Managing	100.36 (14.99)	100.52 (14.36)	100.23 (15.53)	0.18	0.02

*N* = 377. M, mean; SD, standard deviation.

*p < 0.05; **p < 0.01.

Pearson’s correlations (see Table 2) revealed significant positive associations between age and all general developmental domains and EI abilities, with effect sizes ranging from small to large (*r*s = 0.14–0.56, *p*s < 0.05). The strongest correlations were observed between age and the cognitive, communication, and motor domains. By comparison, associations between age and perceiving, understanding, and managing emotions were smaller, although all remained statistically significant.

**Table 2:** Pearson correlations among age and developmental domains and abilities.

Variable	1	2	3	4	5	6	7
1. Age	—						
2. Cognitive	0.56**	—					
3. Communication	0.51**	0.61**	—				
4. Motor	0.50**	0.45**	0.33**	—			
5. Perceiving	0.22**	0.22**	0.22**	0.20**	—		
6. Understanding	0.14**	0.21**	0.21**	0.08	0.12*	—	
7. Managing	0.16**	0.31**	0.25**	0.12*	0.15**	0.12*	—

*N* = 377. *p < 0.05, **p < 0.01.

We then evaluated whether linear or quadratic functions better characterised the associations between age and each developmental outcome. Among the general developmental domains, the linear model explained 30.9% of the variance in cognitive ability, while the quadratic model explained 31.2%, representing an increase of only 0.3 percentage points. For communication ability, both models explained 26.1% of the variance. For motor ability, the quadratic model explained slightly more variance than the linear model (26.0% vs. 24.8%). Associations between age and the EI abilities were smaller. For perceiving emotions, the linear and quadratic models explained 4.0 and 4.1% of the variance, respectively; the corresponding values were 1.5 and 1.6% for understanding and 1.9 and 2.2% for managing. Although all models were statistically significant overall, adding the quadratic term increased explained variance by no more than 1.2 percentage points. The linear models therefore provided the most parsimonious representation of the associations between age and the outcomes examined (Figure 1).

**Figure 1:**
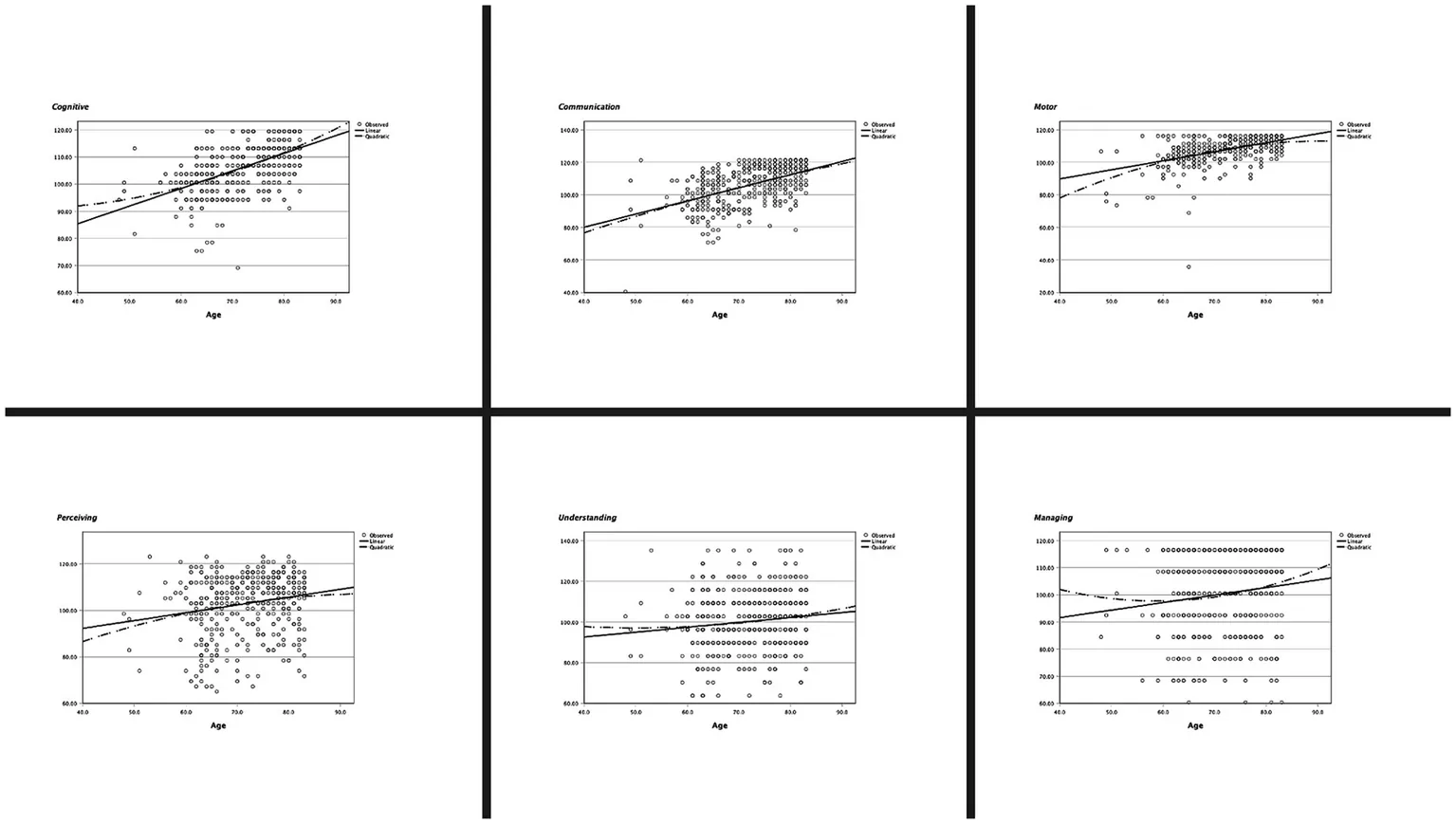
Linear and quadratic associations between age (in months) and scores for the general developmental domains (cognitive, communication, and motor abilities) and emotional intelligence abilities (perceiving, understanding, and managing).

Pearson’s correlations were used to examine the associations between the general developmental domains and EI abilities (see Table 2). The cognitive, communication, and motor domains were positively interrelated. The strongest correlation was between cognitive and communication abilities (*r* = 0.61, *p* < 0.001), followed by cognitive and motor abilities (*r* = 0.45, *p* < 0.001) and communication and motor abilities (*r* = 0.33, *p* < 0.001).

EI abilities were also positively associated with one another, although these correlations were smaller. Perceiving correlated with both understanding (*r* = 0.12, *p* = 0.018) and managing (*r* = 0.15, *p* = 0.003), while understanding was correlated with managing (*r* = 0.12, *p* = 0.017).

The general developmental domains were also positively associated with the EI abilities, with cognitive ability showing the strongest overall pattern of associations. In particular, it was associated with managing (*r* = 0.31, *p* < 0.001), perceiving (*r* = 0.22, *p* < 0.001), and understanding emotions (*r* = 0.21, *p* < 0.001). Communication ability showed a similar pattern, with correlations ranging from 0.21 to 0.25 across the three EI abilities (all *p*s < 0.001). Motor ability was more modestly associated with perceiving (*r* = 0.20, *p* < 0.001) and managing (*r* = 0.12, *p* = 0.022), but was not significantly associated with understanding (*r* = 0.08, *p* = 0.120).

Finally, three hierarchical stepwise regression analyses were conducted to examine the extent to which the general developmental domains explained variance in perceiving, understanding, and managing beyond that accounted for by age and gender. The analyses followed a two-block procedure, with age and gender considered in the first block and motor, communication, and cognitive abilities in the second. Variables within each block were entered using stepwise selection (see Table 3).

**Table 3:** Hierarchical stepwise regression models and final-model coefficients predicting emotional intelligence abilities.

Outcome and step	Model	Predictor	*β*	*p*	*R* ^2^	Δ*R*^2^
*Perceiving*						
Step 1	1	Age	0.215	< 0.001	0.046	0.046
Step 2	2	Gender	0.119	0.018	0.060	0.014
Final model (Step 3)	3	Age	0.119	0.048	**0.081**	**0.021**
		Gender	0.141	0.005		
		Cognitive	0.177	0.004		
*Understanding*						
Step 1	1	Age	0.139	0.007	0.019	0.019
Final model (Step 2)	2	Age	0.047	0.425	**0.044**	**0.024**
		Communication	0.181	0.002		
*Managing*						
Step 1	1	Age	0.155	0.003	0.024	0.024
Final model (Step 2)	2	Age	−0.024	0.690	**0.096**	**0.072**
		Cognitive	0.322	< 0.001		

*N* = 377. Gender was coded as 0 = boys and 1 = girls. Bold values indicate R² and ΔR² for the final model in each analysis.

The first hierarchical stepwise regression analysis examined perceiving. Age entered the model at the first step and accounted for 4.6% of the variance (*R*^2^ = 0.046, *p* < 0.001). At the second step, the addition of gender explained a further 1.4% of the variance (*R*^2^ = 0.060, Δ*R*^2^ = 0.014, *p* = 0.018). Cognitive ability entered at the final step, increasing the total proportion of explained variance to 8.1% (*R*^2^ = 0.081, Δ*R*^2^ = 0.021, *p* = 0.004). In the final model, cognitive ability was a significant positive predictor (*β* = 0.177, *p* = 0.004). Age (*β* = 0.119, *p* = 0.048) and gender (*β* = 0.141, *p* = 0.005) also remained significant predictors, although their contributions were smaller.

The second analysis examined understanding. Age entered the model at the first step and accounted for 1.9% of the variance (*R*^2^ = 0.019, *p* = 0.007). At the second step, the addition of communication ability explained a further 2.4% of the variance, increasing the total proportion of explained variance to 4.4% (*R*^2^ = 0.044, Δ*R*^2^ = 0.024, *p* = 0.002). In the final model, communication ability was a significant positive predictor of understanding (*β* = 0.181, *p* = 0.002), whereas age was no longer significant (*β* = 0.047, *p* = 0.425).

The third analysis examined managing. Age entered the model at the first step and accounted for 2.4% of the variance (*R*^2^ = 0.024, *p* = 0.003). At the second step, the addition of cognitive ability explained a further 7.2% of the variance, increasing the total proportion of explained variance to 9.6% (*R*^2^ = 0.096, Δ*R*^2^ = 0.072, *p* < 0.001). In the final model, cognitive ability was a significant positive predictor of managing (*β* = 0.322, *p* < 0.001), whereas age was no longer significant (*β* = −0.024, *p* = 0.690).

## 4 Discussion

The present study examined age-related patterns in performance-based EI abilities among children aged 4–6 years and their associations with cognitive, communicative, and motor abilities. Overall, the findings partially support the proposed hypotheses. EI abilities were positively and predominantly linearly associated with age, although these associations were weaker than those observed for the general developmental abilities. General developmental abilities were also positively associated with EI, but the multivariable analyses revealed distinct patterns across perceiving, understanding, and managing. Finally, gender differences were limited to perceiving, with girls scoring slightly higher than boys.

The first hypothesis received only partial support. Girls scored higher than boys on perceiving emotion, and gender remained a small but significant predictor in the final model. However, no gender differences emerged for understanding or managing emotions. These findings suggest that the advantage in performance-based EI reported among girls and women during adolescence and adulthood, respectively (Cabello et al., 2016b; Megías-Robles et al., 2024), may not yet extend across EI abilities during early childhood. The absence of a gender difference in managing also contrasts with previous evidence of better emotional regulation among girls (Liao and Lin, 2025). This discrepancy may reflect differences between general emotion-regulation behaviour and performance-based assessments of knowledge about emotion-management strategies. Given the small effect observed, the gender difference in perceiving should be interpreted cautiously.

The findings supported the second hypothesis, which proposed a positive correlation between age and all general developmental domains. This correlation was particularly strong for cognitive, communication, and motor skills, with large effect sizes consistent with existing literature (Rhemtulla and Tucker-Drob, 2011). Additionally, in line with the third hypothesis, age was positively associated with EI abilities. However, the relationships between age and the perception, understanding, and management of emotions were weaker. Moreover, the quadratic models provided only trivial improvements over the linear models, suggesting that age-related differences between 4 and 6 years were most parsimoniously characterised as linear. This finding is consistent with the linear age-related pattern previously reported for EI among adolescent girls (Megías-Robles et al., 2024).

Nevertheless, age was no longer a significant predictor of understanding or managing once we accounted for communication and cognitive functioning. This pattern suggests that the associations between chronological age and these EI abilities may partly reflect age-related improvements in broader developmental resources, consistent with the possibility that EI abilities are embedded within a more general developmental factor (Trudel et al., 2026). For perceiving, however, age retained a small independent association. As perceiving represents a more foundational ability within the ability model, it may follow a somewhat distinct developmental trajectory. It may also be shaped by personal and social factors that are not fully captured by measures of general developmental abilities (Mayer and Caruso, 2025).

The fourth hypothesis was broadly supported at the bivariate level but only partially supported in the multivariate models. Cognitive, communication, and motor abilities were generally positively associated with EI, although the associations were modest, and the relationship between motor ability and understanding was not statistically significant. Importantly, the regression models revealed ability-specific patterns. Communication ability independently predicted understanding, whereas cognitive ability predicted perceiving and, most strongly, managing. These findings are consistent with the possibility that interpreting emotional information draws partly on language and communication resources, whereas identifying emotions and selecting appropriate emotion-management strategies may place greater demands on broader cognitive processes (Mayer and Caruso, 2025; Yu et al., 2023). Motor ability made no independent contribution once age, gender, and other developmental domains were considered. Its bivariate associations with EI may therefore reflect variance shared with age and cognitive ability (Bao et al., 2024; Van Der Fels et al., 2015). Future longitudinal studies encompassing a wider age range should further clarify the relationships between EI abilities and general developmental abilities.

These differentiated patterns reinforce the conceptualisation of EI as a set of related but distinct abilities, as proposed by the ability model of EI (Mayer and Salovey, 1997; Mayer et al., 2016). Indeed, the relatively small correlations among perceiving, understanding, and managing further suggest that these abilities are not interchangeable during early childhood. The findings therefore extend integrative perspectives on child development (Denham, 1998) by indicating that emotional abilities are embedded within broader developmental processes while retaining a degree of specificity (Trudel et al., 2026). From an educational perspective, interventions may benefit from combining emotional content with developmentally appropriate cognitive and language-based activities, as reflected in existing EI intervention programmes (Brackett et al., 2019; Cabello et al., 2016a).

Certain limitations should be acknowledged. First, the cross-sectional design precludes conclusions about intraindividual developmental change or the direction of the observed associations. Second, the restricted age range may have limited the variability needed to detect more complex age-related patterns. Third, the reliability estimate for understanding is lower than that of the other EI abilities, which may affect how findings involving this dimension are interpreted, as it could weaken observed associations. Fourth, the modest variance explained (4.4–9.6%) underscores the complex, multifactorial nature of EI in early childhood, where unmeasured family, social, educational, and temperamental factors inevitably play key roles. However, a modest proportion of explained variance does not diminish the study’s theoretical or practical relevance. To enhance predictive capacity, future research should integrate specific contextual variables—such as parenting practices, parent–child emotional communication, parental emotional competencies, and broader social and educational environments. Expanding the model to complement these developmental dimensions will establish a more comprehensive framework for understanding how emotional abilities emerge and evolve during these formative early years. Finally, hierarchical stepwise regression is a data-driven approach; consequently, the identified models should be replicated in independent samples using theoretically specified predictors.

Despite these limitations, the study provides new evidence from a performance-based measure of EI and supports an integrative, multidimensional understanding of emotional development during early childhood. Overall, the findings highlight the potential value of integrating emotional and cognitive skill development within early educational settings. Notably, the stronger associations between cognitive functioning and the ability to perceive and manage emotions suggest that activities targeting these EI abilities may benefit from incorporating developmentally appropriate cognitive training components. For example, emotion recognition activities could require children to direct their attention and discriminate between relevant facial or contextual cues. Similarly, tasks could present everyday emotional situations where children are asked to consider different emotion-management strategies, anticipate their potential consequences, and select an appropriate response. Given the correlational nature of the current study, these practical applications represent promising avenues for future intervention research rather than established protocols. Similarly, the association between communication ability and understanding emotions suggests that language-rich, interactive activities represent a promising educational approach for fostering this EI ability, a finding that warrants examination in future intervention studies. Ultimately, such approaches could support adaptive socio-emotional functioning, emotional regulation, and healthy development during childhood.

## Data Availability

The data supporting the findings of this study are available from the corresponding author upon reasonable request, subject to applicable ethical and data-protection restrictions.
